# Precise Image Color Correction Based on Dual Unmanned Aerial Vehicle Cooperative Flight

**DOI:** 10.1016/j.plaphe.2025.100101

**Published:** 2025-09-05

**Authors:** Xuqi Lu, Jiayang Xie, Jiayou Yan, Ji Zhou, Haiyan Cen

**Affiliations:** a State Key Laboratory for Vegetation Structure, Function and Construction (VegLab), College of Biosystems Engineering and Food Science, Zhejiang University, Hangzhou, 310058, PR China; b Key Laboratory of Spectroscopy Sensing, Ministry of Agriculture and Rural Affairs, Hangzhou, 310058, PR China; c Yuan Longping High-Tech Agriculture Co., Ltd, Changsha, 410128, PR China; d Cambridge Crop Research, National Institute of Agricultural Botany (NIAB), Cambridge, CB3 0LE, UK

**Keywords:** Color correction, Color measurement, UAV imaging, Cooperative flight, Lighting variation

## Abstract

Color accuracy and consistency in remote sensing imagery are crucial for reliable plant health monitoring, precise growth stage identification, and stress detection. However, without effective color correction, variations in lighting and sensor sensitivity often cause color distortions between images, compromising data quality and analysis. This study introduces a novel in-flight color correction approach for RGB imagery using cooperative dual unmanned aerial vehicle (UAV) flights integrated with a color chart (CoF-CC). The method employs a master UAV equipped with an RGB camera for image acquisition and a synchronized secondary UAV carrying a ColorChecker (X-Rite) chart, ensuring persistent visibility of the chart within the imaging field of the master UAV for the calculation of a color correction matrix (CCM) for in-flight image correction. Field experiments validated the method by analyzing cross-sensor color consistency, assessing color measurement accuracy on field-grown rice leaves, and demonstrating its practical applications using rice maturity estimation as an example. The results indicated that the CCM significantly enhanced color accuracy, with a 66.1 ​% reduction in the average CIE 2000 color difference (ΔE), and improved color consistency among the six RGB sensors, with a 70.2 ​% increase in the intracluster distance. CoF-CC subsequently reduced ΔE from 18.2 to 5.0 between the corrected rice leaf color and ground-truth measurements, indicating that the color differences were nearly perceptible to the human eye. Moreover, the corrected imagery significantly enhanced the rice maturity prediction accuracy, improving the R^2^ from 0.28 to 0.67. In summary, the CoF-CC method standardizes RGB images across diverse lighting conditions and sensors, demonstrating robust performance in color analysis and interpretation under open-field conditions.

## 1 Introduction

In modern agricultural production and breeding research, precise color acquisition in plants, such as leaves, flowers, and fruits, has become increasingly important because it is related to various biotic and abiotic stresses in plants, such as diseases [[1], [2], [3]], water deficiency [4,5], and nutrient deficiency [[6], [7], [8]]. Moreover, color parameters are typically used as key indicators in variety selection to identify new cultivars that meet market demands and consumer preferences. Traditionally, plant color traits are evaluated by visual inspection, which is not only time-consuming and inaccurate but also infeasible when applied at field scales or for numerous samples.

In recent years, the use of unmanned aerial vehicles (UAVs) equipped with high-resolution RGB cameras for low-altitude remote sensing has become increasingly prevalent in agriculture, transforming precision breeding and crop monitoring practices [[9], [10], [11]]. Compared with ground-based measurements, which are limited in coverage and throughput, and satellite imagery, which lacks sufficient spatial resolution and temporal flexibility, UAVs offer high spatial resolution, flexibility, and wide coverage, thereby enabling the rapid acquisition of large-scale farmland imagery and providing an efficient means of color measurement [[12], [13], [14]]. Additionally, compared with traditional manual sampling methods or costly manned aerial surveys, UAV-based remote sensing provides a more cost-effective solution, significantly lowering operational expenses while remaining easily accessible for routine agricultural applications. Nevertheless, UAV-acquired images in open-field environments are strongly influenced by diverse sensor characteristics, variations in flight altitude, lighting conditions, imaging angles, and camera settings, which often result in color distortions and inconsistent radiometric quality both within the same flight mission and between different experiments [15,16]. The significant variation introduced by environmental factors was demonstrated by Tocci et al. [17], whose study illustrated discrepancies in color through repeated field imaging acquisitions and orthomosaic reconstructions across different fields and times. Consequently, without effective color correction methods, UAV-derived plant color measurements inherently suffer from poor color consistency and low color accuracy, complicating data reuse, sharing, and quantitative analysis and severely limiting their reliability for precise crop management and breeding decisions.

Recent studies have attempted to address the issue of color inconsistency caused by variations in illumination or camera parameters within the same flight mission by employing image color feature transfer methods. Some of these approaches involve either manual or automatic selection of a reference image as a color standard for flight and the use of simple machine learning or deep learning algorithms to extract color features and determine transformation rules, thereby mapping the color style of other images onto this reference [[18], [19], [20]]. Li et al. [21] and Lin et al. [22] conducted statistical analysis across the entire image set acquired in one flight campaign by using optimization algorithms to eliminate global and local radiometric differences, aiming to achieve more consistent color representations during image mosaicking. Although such methods can improve color consistency within a single flight mission, their effectiveness is highly dependent on the representativeness of the selected reference image, thereby increasing uncertainty under conditions of dynamic environmental illumination. Furthermore, these studies often lack an evaluation of image color accuracy, raising concerns regarding the practical accuracy and reliability of their methods in agricultural applications. A widely used method to ensure image color accuracy is the use of standard color reference charts, such as the ColorChecker of X-Rite, which are widely used in photography [23,24], printing [25], and dentistry [26,27]. These charts allow researchers to compare field-acquired images with standard reference color patches for color correction. In agricultural research, color charts are typically placed alongside crops within the image frame, and postprocessing color correction algorithms are subsequently applied to achieve color standardization [28,29]. However, this approach faces practical challenges in UAV-based remote sensing applications. Placing only a single color reference chart at a fixed location results in limited coverage across UAV images, which makes it difficult to adequately correct color variations caused by changing illumination conditions and camera parameters throughout the flight. Conversely, embedding color charts in every imaging view requires substantial effort and time, potentially obstructing or negatively affecting crop imaging quality.

In this study, a dual-UAV cooperative flight method with color chart integration (CoF-CC) for in-flight UAV image color correction is introduced. This approach enhances the accuracy and consistency of crop color data acquisition under dynamic open-field lighting conditions and facilitates reliable data reuse across different UAV platforms, thereby improving data-driven decision-making in crop management. The main objectives of the study are (1) to develop a system that ensures that each UAV image contains a color reference by employing dual-UAV cooperative flights, enabling in-flight color correction under dynamic lighting; (2) to evaluate the efficiency of the CoF-CC method in color correction across different imaging sensors and in-flight imagery, as well as its accuracy in predicting rice leaf colors in comparison to ground-truth measurements; and (3) to demonstrate the practical value of precise crop color measurements in agricultural applications, exemplified by using crop color as an indicator to estimate rice maturity dates.

## 2 Materials and methods

### 2.1 Proposed workflow for in-flight color correction in the field

#### 2.1.1 Dual-UAV cooperative flight

**Fig. 1:**
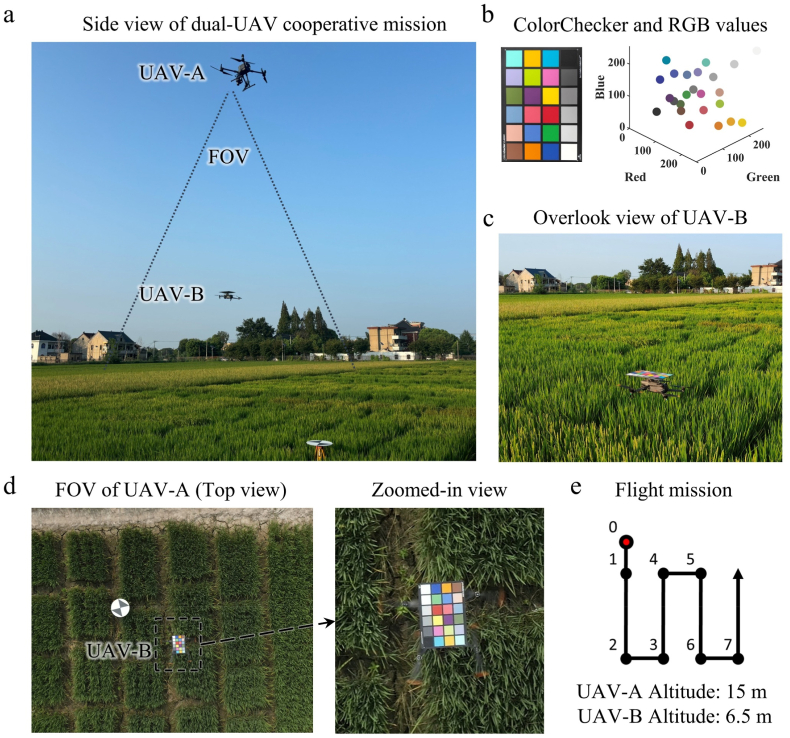
(a) Side view of the dual-UAV cooperative flight. (b) ColorChecker and its RGB values visualized in 3D space. (c) UAV-B carrying ColorChecker during flight, shown in the overhead view. (d) Representative image captured by UAV-A. (e) Flight mission schematic, where black points represent standard waypoints and the red point indicates the starting point for mission synchronization preparation.

Two UAVs, designated UAV-A and UAV-B, were organized into a coordinated UAV flight formation. In all the experiments, UAV-A and UAV-B were flown at altitudes of 15 ​m and 6.5 ​m, respectively (Fig. 1a). UAV-A served as a standard imaging platform for proximal remote sensing and was responsible for acquiring orthophoto images of the field. UAV-B carried a ColorChecker (ColorChecker Classic, X-Rite, Michigan, USA), as shown in Fig. 1b, and flew in synchrony with UAV-A to ensure that the ColorChecker remained within the camera field of view of UAV-A during image acquisition. The ColorChecker was securely attached to the top of UAV-B using double-sided foam tape, as shown in Fig. 1c. The stability of the attachment was thoroughly validated in preliminary trials to ensure that ColorChecker remained level throughout the flight. Since both UAVs followed the same flight path, UAV-B consistently maintained a central position in the images acquired by UAV-A, with ColorChecker occupying a size of approximately 180 ​× ​270 pixels in the images. A clear view of ColorChecker from UAV-A's nadir perspective is shown in Fig. 1d. To determine the difference in altitude between UAV-A and UAV-B, preliminary tests were conducted with ColorChecker positioned at vertical distances ranging from 6 ​m to 10 ​m beneath the UAV-A camera. As shown in Table S1, the average RGB values of the 24 color patches varied by less than 3 ​%, indicating a negligible impact on color consistency. Considering both the imaging field of view and the rotor downwash effect on the crop canopy, the altitude of UAV-B was set to 6.5 ​m when UAV-A operated at 15 ​m.

The coordinated flight missions of UAV-A and UAV-B were achieved by generating DJI WayPoint Markup Language route files using custom R code (v4.1.2) combined with the DJI Cloud API (v1.4.0; DJI; Shenzhen, China). To address the time difference between the two UAVs reaching their starting points from the take-off position, an additional waypoint (waypoint 0) was added 1 ​m behind the first waypoint in the flight mission, as shown in Fig. 1e. If either UAV arrived at Waypoint 0 ahead of the other, its mission would pause until the other UAV reached the same waypoint, ensuring synchronization for the remainder of the flight. Minor adjustments were made to UAV-B during the flight mission to mitigate occasional speed discrepancies caused by external wind conditions. The flight speed of both UAVs was set to 1.2 ​m/s, with side and forward overlaps of 80 ​%. These parameters were easily adjustable within the customized code.

To present the variations in light conditions during a UAV flight, the brightness value, which was calculated as the average of the R, G, and B channels of the 18 ​% gray patch (the 22nd patch of ColorChecker), was used as a reference. The 18 ​% gray patch reflects approximately 18 ​% of the incident light, serving as a standard reference for brightness calibration [30]. To minimize the influence of varying camera parameters on the brightness measurements, the brightness values were normalized to the following standard conditions: ISO 100, a shutter speed of 1/100 ​s, and an aperture of f/8. The normalization process was performed using the following formula:

(1) $$\begin{array}{c} L_{std}=L_{raw}\times \frac{{ISO}_{ref}}{{ISO}_{raw}}\times \frac{t_{ref}}{t_{raw}}\times {\left(\frac{N_{raw}}{N_{ref}}\right)}^{2} \end{array}$$

where $L_{std}$ represents the brightness after normalization; $L_{raw}$ is the initial brightness value; ${ISO}_{ref}$ and ${ISO}_{raw}$ are the reference and initial ISO values, respectively; $t_{ref}$ and $t_{raw}$ are the reference and initial shutter speeds, respectively; and $N_{ref}$ and $N_{raw}$ are the reference and initial aperture values, respectively.

#### 2.1.2 Image correction and mosaicking with masked ColorChecker

**Fig. 2:**
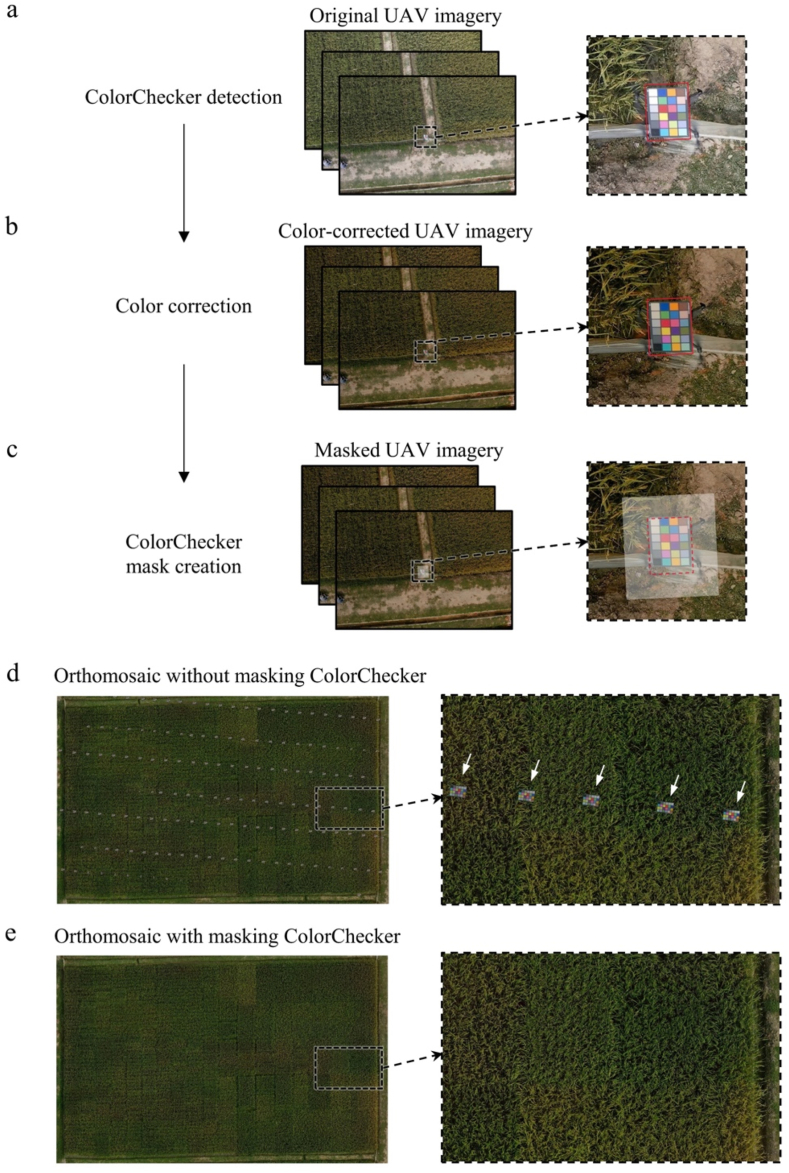
Workflow for UAV image color correction and mosaicking. (a) Detection of ColorChecker boundary points in UAV imagery. (b) Calculation of the color correction matrix (CCM) for image color correction. (c) Mask generation based on the outward expanded ColorChecker boundary points. (d) Orthomosaic generation without masking. (e) Orthomosaic generation with masking. The white arrows in (d) indicate the interference of ColorChecker in the orthomosaic.

As a single UAV flight mission generates hundreds of images for further processing, we constructed an automated workflow for generating a color-corrected field orthomosaic using original UAV imagery as input, as illustrated in Fig. 2. First, a typical UAV image containing a clearly visible ColorChecker was selected from the flight and cropped to obtain the ColorChecker region, which served as a template. The scale-invariant feature transform (SIFT) [31] algorithm was subsequently used to extract keypoints and descriptors from both the template and each target image. To establish correspondences, the brute-force matcher (BFMatcher) [32] was applied to identify reliable matches between the two sets of keypoints. Based on the matched point pairs, a homography transformation matrix was estimated to accurately locate the ColorChecker region in each target image (Fig. 2a). For the datasets acquired in this study, all the images were manually inspected, and no misdetections were detected. After detecting the ColorChecker in each image, we used the colorChecker function in MATLAB 2021a (MathWorks, Inc., Natick, MA, USA) to accurately locate the 24 color patches. The function first computes the geometric transformation matrix based on the correspondence between the four detected corner coordinates of ColorChecker in the image and the known physical corner coordinates of a standard reference ColorChecker. Using this transformation, the physical center positions of the 24 color patches were projected into the image space. Approximately 80 ​% of the patch area around each projected patch center was used to compute the average RGB value, which eliminated the influence of boundary noise and neighboring regions. Finally, color correction matrix (CCM) computation, image color correction, and color error calculation were performed on each image (Fig. 2b). The detailed implementation of these methods is provided in Section 2.2.

The generation of orthomosaic was achieved following the standardized pipeline in Metashape Professional (v1.8.0; Agisoft, LLC, St. Petersburg, Russia). If images containing ColorChecker were directly mosaicked, the algorithm would treat the ColorChecker region as part of the foreground; this would result in the ColorChecker appearing in the final orthomosaic, thereby interfering with image appearance and color extraction (Fig. 2d). To resolve this issue, after color correction, a mask was applied to the ColorChecker regions based on the identified boundaries. The mask size was adjusted by extending its length by 30 ​% in each direction and its width by 50 ​% in each direction to ensure full coverage (Fig. 2c). This adjustment accounts for the dimensions of UAV-B, including its arms and rotors, which are larger than those of ColorChecker itself. The original ColorChecker area was approximately 270 ​× ​180 pixels, and the final mask covered an area of approximately 450 ​× ​360 pixels. In this study, the UAV flight was designed with 80 ​% forward and side overlaps; given the image resolution of 8192 ​× ​5460 pixels, this resulted in an overlap of approximately 4368 pixels in the forward direction and 6554 pixels in the side direction. These overlaps are significantly larger than the masked area, ensuring that the image information missing due to the ColorChecker mask can be effectively supplemented by adjacent images. As a result, the mosaicking process with the designed masks produced field orthomosaics without interference from the ColorCheckers, as shown in Fig. 2e.

### 2.2 ColorChecker and color correction matrix

A color correction method based on ColorChecker and the CCM was applied to correct the original images. ColorChecker, containing 24 meticulously designed RGB color patches, is depicted along with the three-dimensional distribution of the RGB values under the CIE D65 standard illuminant in Fig. 1a. The CCM, a commonly used linear mapping method for color correction, uses the known standard RGB values of the ColorChecker patches as reference data [[33], [34], [35]]. It is determined by minimizing the error between the device-acquired RGB patch values and the reference values. The derived CCM is then used to interpolate and correct the values of other colors that are not directly sampled.

The mathematical principle of the CCM is described as follows. The standard RGB values of the ColorChecker, denoted as *C*, are known and represented as a 24 ​× ​3 matrix. The observed RGB values of the ColorChecker obtained from the image $I_{O}$, denoted as A, are typically a 24 ​× ​3 matrix. To better capture nonlinear color shifts caused by imaging devices and other factors, a bias term is introduced, resulting in A being expanded to a 24 ​× ​4 matrix. The CCM matrix M is then calculated using the process shown in Equations (2), (3).

(2) $$\begin{array}{c} C=A\times M \end{array}$$

Based on principles of linear algebra, the CCM matrix M is calculated as follows:

(3) $$\begin{array}{c} M={\left(A^{T}\times A\right)}^{-1}\times A^{T}\times C \end{array}$$

Once the CCM matrix M is determined, it is applied to correct the other pixels in the image I. The image I, originally represented as a 3D matrix with dimensions w×h×3, is first flattened into a 2D matrix $I_{O}^{\prime }$ of size (w×h)×3. A constant value of 1 is then appended to the RGB values of each pixel as a bias term, forming a new 2D matrix $I_{O}^{\prime \prime }$ with dimensions (w×h)×4. Using Equation (3), the corrected 2D matrix $I_{C}^{\prime }$ with dimensions (w×h)×3 is obtained. Finally, $I_{C}^{\prime }$ is reshaped back into its original image format as $I_{C}$, with dimensions (w×h)×3.

(4) $$\begin{array}{c} I_{C}^{\prime }=I_{O}^{\prime \prime }\times M \end{array}$$

The CIE 2000 color difference formula (CIEDE2000) was adopted as the evaluation metric for color error in this study. CIEDE2000, recommended by the International Commission on Illumination (CIE), provides a more accurate representation of human visual perception of color differences. To calculate this metric, the RGB images were converted into the CIE 1976 (L∗, a∗, b∗) color space, commonly referred to as CIELAB or simply LAB. The LAB values of the region of interest (ROI) were then extracted and compared with the standard LAB values under the D65 illuminant. The detailed calculation formula can be found in Luo et al. [36]. For simplicity, ΔE is used to denote CIEDE2000.

Owing to variations in natural illumination, certain color patches in the captured ColorChecker images may occasionally become overexposed. To ensure accuracy, any color patches with any RGB channel value exceeding 250 were excluded from the calculation of the CCM M. Additionally, a sensitivity analysis was conducted by systematically reducing the number of color patches used in the color correction process to evaluate the impact of the addition of the number of patches on the correction accuracy.

To simulate color distortions resulting from varying imaging environments and sensor responses, random noise was introduced to the RGB channels of the original UAV imagery. Each channel was randomly attenuated within a range of 0.5–1.0 to prevent overexposure of ColorChecker color patches. Image color correction and ΔE evaluation were then performed as previously described.

### 2.3 Color corrections for different camera models

Six UAV camera models were compared in this study to evaluate the effectiveness of the CCM method in improving color consistency. These included three cameras integrated into the DJI Mavic 3 Pro (Mavic 3 Pro, DJI, Shenzhen, China): the wide-angle Hasselblad camera (24-mm equivalent focal length), the medium telephoto camera (70-mm equivalent focal length), and the telephoto camera (166-mm equivalent focal length), referred to as M3 Wide, M3 M-Tele, and M3 Tele, respectively. Additionally, the DJI Mavic 3T (Mavic 3T, DJI, Shenzhen, China) integrated wide-angle camera (24-mm equivalent focal length) is referred to as M3T; the DJI Zenmuse P1 (Zenmuse P1, DJI, Shenzhen, China) paired with a 35 ​mm lens is referred to as P1; and the Phase One iXM-100 (iXM-100, Phase One, Copenhagen, Denmark) paired with an 80-mm RSM lens (Phase One, Copenhagen, Denmark) is referred to as Phase. The UAV platforms and detailed camera specifications are listed in Table 1. The experiments were conducted on a clear afternoon with uniform illumination. Each of the six cameras, mounted on the corresponding UAV platforms described in Table 1, captured images of a ColorChecker placed adjacent to a rice field. During image acquisition, the cameras were set to automatic white balance and manual exposure, with the exposure settings adjusted to minimize overexposure of the ColorChecker patches.

**Table 1:** UAV Platforms and specifications of the six cameras.

UAV platform	Camera model	Sensor size	Image resolution (pixel)	Focal Length (mm)
DJI Mavic 3 Pro	M3 Wide	4/3 CMOS	5280 ​× ​3956	24
	M3 M-Tele	1/1.3″ CMOS	4032 ​× ​3024	70
	M3 Tele	1/2″ CMOS	4000 ​× ​3000	166
DJI Mavic 3T	M3T	1/2" CMOS	8000 ​× ​6000	24
DJI Matrice 300 RTK	P1	35.9 ​mm ​× ​24 ​mm	8192 ​× ​5460	35
	Phase	43.9 ​mm ​× ​32.9 ​mm	11664 ​× ​8750	80

Note: M3 Wide refers to the wide-angle Hasselblad camera integrated into the DJI Mavic 3 Pro, whereas M3 M-Tele and M3 Tele represent the medium telephoto and telephoto cameras of the DJI Mavic 3 Pro, respectively. M3T denotes the integrated wide-angle camera of the DJI Mavic 3T. Additionally, P1 refers to the Zenmuse P1 camera of DJI, and Phase indicates the Phase One iXM-100 camera. The column Focal Length represents either the physical focal length of the lens or its equivalent focal length in a 35 ​mm format.

After obtaining six images of the ColorChecker captured by different cameras over the rice field, the four corner points of the ColorChecker were manually identified. The 24 color patches were then automatically segmented using the colorChecker function, and their RGB values were extracted. The CCM for each image was computed, and the corrected images were generated by applying the CCM. Based on the RGB values of the color patches extracted from both the original and corrected images, the ΔE values for the original and corrected color patches were calculated. The improvement in ΔE was used to evaluate the effectiveness of the CCM method for each camera.

To evaluate the performance consistency of the CCM method across different cameras, this study employed the calculation of the average intracluster distance in the LAB color space. For each color patch, the distribution of the derived color values across different cameras in the LAB color space was treated as a cluster. The consistency of the original and corrected images was assessed by measuring the average Euclidean distance of points within each cluster from its centroid. The formula for the average intracluster distance is defined as follows:

(5) $$\begin{array}{c} D_{avg,i}=\frac{1}{n_{k}}\sum_{k=1}^{n_{k}}\sqrt{{\left(L_{i,k}-{\bar{L}}_{i}\right)}^{2}+{\left(A_{i,k}-{\bar{A}}_{i}\right)}^{2}+{\left(B_{i,k}-{\bar{B}}_{i}\right)}^{2}} \end{array}$$

where *i* represents the *i*-th color patch, *k* represents the *k*-th camera, and $n_{k}$ ​= ​6, indicating that there are six camera models. ${\bar{L}}_{i}$, ${\bar{A}}_{i}$, and ${\bar{B}}_{i}$ denote the average values of the LAB channels for the *i*-th color patch across all the cameras. $D_{avg,i}$ represents the average intracluster distance for the *i*-th color patch.

### 2.4 The validation of the CoF-CC method

#### 2.4.1 Comparison with ground-truth leaf color measurements

The field trial was conducted at the breeding site of Longping High-Tech (18°28′59″N, 110°3′3″E) in Lingshui, Hainan Province, China, during the grain-filling stage of rice on April 22, 2024, as shown in Fig. S1. Rice sowing was conducted in late December, and transplanting occurred in late January. The trial consisted of 145 plots in total, including 35 larger breeding plots with a size of 5 ​m ​× ​2.7 ​m and 110 smaller breeding plots with a size of 2.5 ​m ​× ​2.7 ​m. Both plot types were arranged with a spacing of 16.7 ​cm ​× ​26.7 ​cm and an interplot spacing of 33 ​cm ​× ​33 ​cm.

To obtain ground-truth leaf color measurements, leaves were randomly sampled from 30 locations in the field. At each sampling point, five leaves were collected as replicates, and the real-time kinematic (RTK) coordinates were recorded using a DJI Mavic 3T to determine the precise location of each sampling point, as shown in Fig. S1. The collected leaves were quickly transferred to the field ridge, where the color values were measured using a colorimeter (WR-10QC; ShenZhen Wave Optoelectronics Technology Co., Ltd., Shenzhen, China). Measurements were taken on the upper, middle, and lower sections of both the adaxial and abaxial surfaces of each leaf, with the leaves fully flattened during measurement. The built-in light source of the colorimeter was set to D65. The average LAB values were calculated to represent the ground truth color at each sampling point.

Based on the RTK coordinates of the leaf sampling points, their corresponding locations on the field orthomosaic could be identified. A subimage of 1200 ​× ​1200 pixels was cropped around each sampling point. This process was repeated for both the original and corrected orthomosaics, yielding a total of 60 subimages. Using LabelMe 5.0.1 (https://github.com/labelmeai/labelme), 100 complete rice leaves in each subimage were randomly and manually labeled. The average LAB color of these 100 labeled rice leaves represented the leaf color from the UAV imagery for that sampling point. For both the original and corrected images, the ΔE values were calculated against the ground truth from the colorimeter. To ensure a fair comparison between the original and corrected subimages, the same set of 100 annotated leaves in each corresponding pair of subimages was used.

#### 2.4.2 Consistency between predicted and manually labeled leaf areas

To further evaluate the effectiveness of the CoF-CC method, this study compared the agreement between the predicted leaf area and the actual leaf area. The predicted leaf area was defined as regions in the original and corrected images where the color error (ΔE) relative to the ground-truth leaf color (measured in LAB values using a colorimeter) fell within a threshold (ΔE below 10). A higher degree of overlap between the predicted leaf area and the actual leaf area indicated greater color accuracy in the images. This comparison was conducted across 30 sampling plots, corresponding to the leaf sampling locations described earlier.

To quantify the overlap, the manually labeled leaf area was used as the ground-truth region. A pixelwise logical AND operation was performed between the predicted leaf area and the ground-truth region to extract the overlapping area. The area of this overlap was then divided by the total area of the ground-truth region to calculate the overlap ratio, which served as a quantitative metric for evaluating the consistency between the predicted and actual leaf areas.

### 2.5 Crop field color visualization and rice maturity prediction

To further demonstrate the value of the CoF-CC method in remote sensing data standardization and its field application, color variation maps for breeding plots were generated, and a correlation analysis between rice color and maturity date (days from sowing to maturity) was conducted. First, ArcMap (v10.8; Esri, Redlands, CA, USA) was used to manually extract individual plots from the experimental field and compute the mean RGB values for each plot. These mean RGB values were then mapped to align with the original field layout, enabling breeders to easily observe the color differences between the breeding materials. Additionally, in 18 breeding plots with available ground-truth data, linear regression analysis was performed to assess the relationships between the rice maturity dates and the normalized (R ​+ ​B) values derived from both the original and color-corrected images.

## 3 Results

### 3.1 Color correction across camera models

**Fig. 3:**
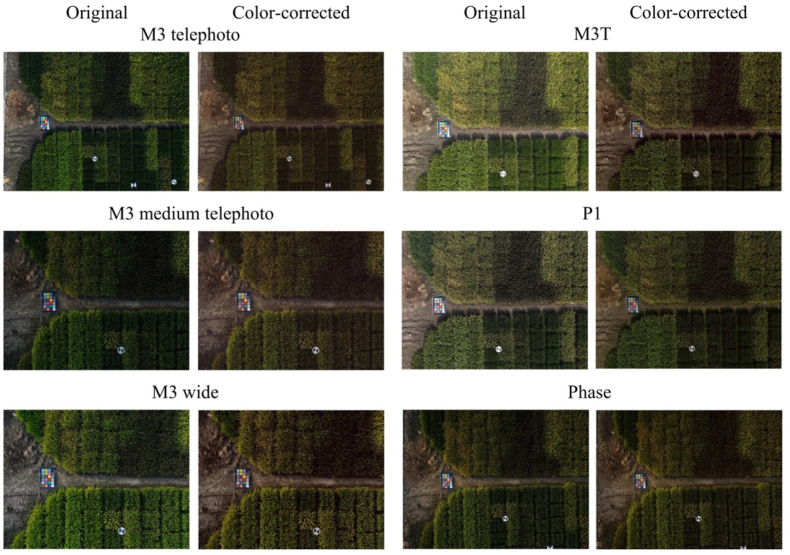
Comparison of original and color-corrected images captured by six different camera models.

**Fig. 4:**
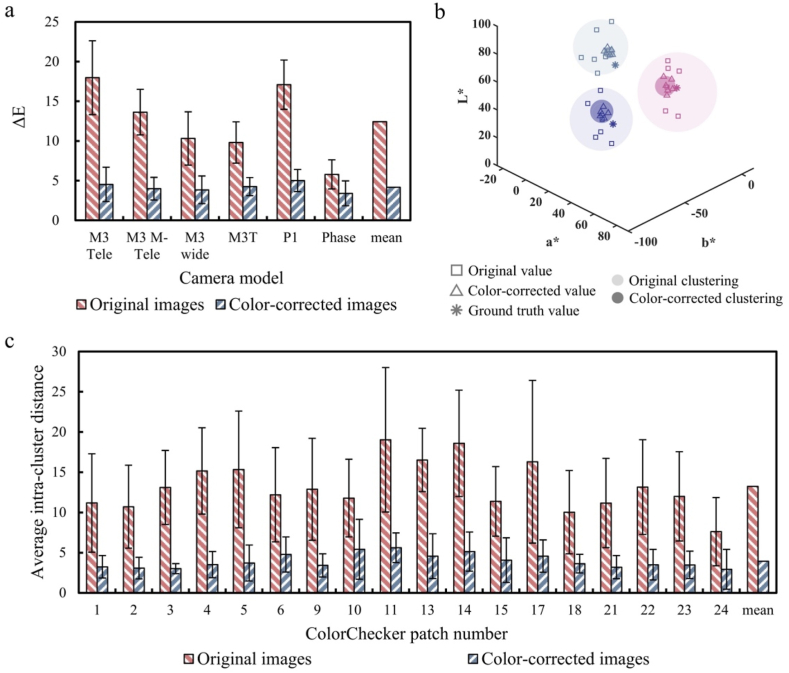
(a) ΔE for ColorChecker of original and corrected images across six camera models. (b) LAB distribution of three selected ColorChecker patches from original and color-corrected images: the squares represent the LAB values for each patch from the original images across all six cameras; the triangles represent the LAB values for color-corrected images across six cameras; and the asterisks indicate the true LAB value of each patch. Light and dark circles show the spread of LAB values for each patch across different cameras in the original and color-corrected images, respectively. Different colors represent different patches within the panel. (c) Intracluster distance for original and color-corrected ColorChecker patches across six cameras, where the intracluster distance is defined as the average distance between the LAB values of each patch from different cameras and the centroid of that patch.

The original and corrected images, which include both the ColorChecker and the rice field, captured by six different camera models, are presented in Fig. 3. Significant brightness and color discrepancies were observed in the original images from the different cameras, but these discrepancies were largely alleviated after color correction. In the original images, the measured ΔE for the ColorChecker patches ranged from 5.8 to 18.0 (Fig. 4a), which was reduced to a range of 3.4–5.0 following color correction, indicating an average 66.1 ​% improvement in color accuracy.

An examination of the LAB color distributions in 3D space revealed significantly smaller clusters of extracted color values for color-corrected images (Fig. 4b). The corrected patch colors were not only more tightly clustered but were also positioned closer to the ground truth color values than the colors measured from the original patches. The effectiveness of the color correction method was further validated by calculating the intracluster distances for each color patch across all six different camera models in the LAB color space. The average intracluster distance decreased from 13.2 in the original images to 3.9 after color correction, representing a 70.2 ​% improvement in color consistency (Fig. 4c).

### 3.2 Accuracy assessment of the CoF-CC method

**Fig. 5:**
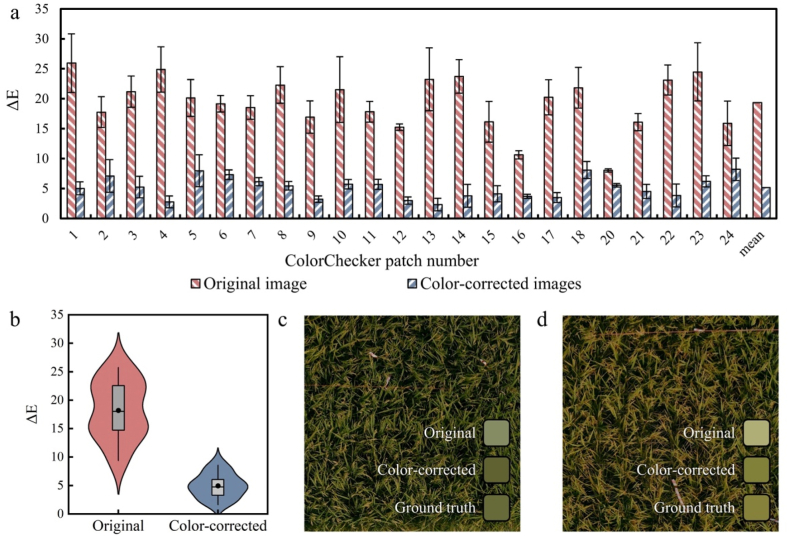
(a) Assessment of color accuracy errors in original and color-corrected images, evaluated using ColorChecker patches; (b) assessment of leaf colors sampled from field plots. (c, d) Images of two representative field plots after color correction. The inserted color blocks represent the colors obtained from the original images, color-corrected images, and ground truth measurements.

A summary of the performance evaluation for the CoF-CC method, validated using ColorChecker color patches and leaf samples obtained from a large-scale field flight mission, is provided in Fig. 5. The ΔE values, obtained from temporally and spatially diverse image sets, initially ranged from 8.0 to 25.9 across the color patches (Fig. 5a). Following color correction, these values were significantly reduced to a range of 2.3–8.2, indicating a 73.6 ​% improvement in color accuracy. Furthermore, the CoF-CC method exhibited robust performance with color-cast images, effectively reducing artificial color distortions to an average ΔE value of 7.1 following the correction process (Fig. S2).

Comparisons of leaf colors between the original images and ground truth measurements at 30 sampling points revealed a substantial initial color discrepancy, with a mean ΔE value of 18.2 (Fig. 5b). The implementation of color correction significantly reduced the mean ΔE value to 5.0, corresponding to a 72.7 ​% reduction in color error. The ΔE values measured for color-corrected ColorChecker patches and leaf samples were closely aligned, demonstrating the consistency and robustness of the CoF-CC method across diverse measurement targets. The visual outcomes of the color correction method are shown in Fig. 5c and d, with two representative plots with plants at different stages of ripening. For comparison, the original images are presented in Fig. S3. Visual assessment demonstrated that color variations were substantially minimized following color correction, enabling more reliable discrimination of phenological differences.

### 3.3 Alignment of predicted leaf area in UAV imagery with manual labeling

**Fig. 6:**
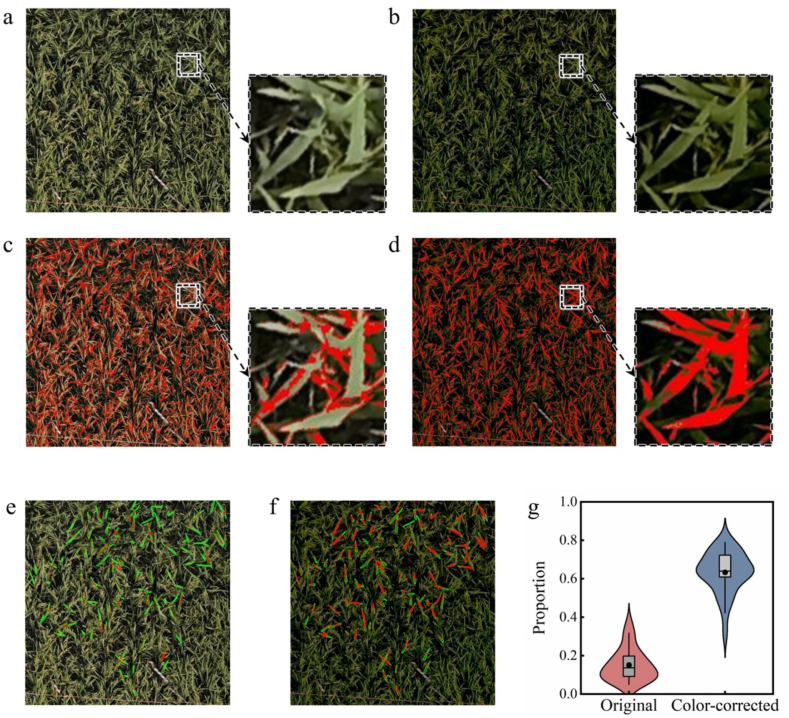
(a, b) Original and color-corrected images for one plot as an example, showing leaf details. (c, d) Original and color-corrected images for the same plot, with red highlighting regions where the difference in color of ΔE was below a threshold of 10 compared with the ground-truth leaf color sampled from field plots. (e, f) Original and color-corrected plot images with low color difference regions in red are displayed only within a set of 100 manually annotated leaves, indicated in green for clarity. (g) Proportion of annotated leaves with ΔE ​< ​10 in the original and color-corrected images. Red points indicate areas where ΔE < 10 compared with the ground truth of the leaf color sampled in the field plots.

Given that leaf areas within UAV imagery can be clearly distinguished based on color, the efficacy of the CoF-CC method was evaluated by mapping ground-truth leaf color onto both original and color-corrected images, followed by a comparative analysis of their overlapping regions. The original image of a representative plot and its color-corrected version are presented in Fig. 6a–b, respectively. In the original image, the segmentation of areas closely matching the ground-truth leaf color showed poor alignment with the actual leaf shape (Fig. 6c). The segmented regions were scattered and were located mainly along the edges and bases of the leaf positions. In contrast, applying the same segmentation criteria to the color-corrected image revealed an organized pattern with a high degree of alignment to actual leaf areas based on visual assessment (Fig. 6d).

A quantitative analysis was conducted to evaluate the overlap between the segmented leaf area generated using ground truth color and the manually labeled leaf area. The degree of overlap was significantly greater in color-corrected images, as illustrated in a representative example (Fig. 6e–f), indicating reduced color discrepancy in the corrected images. Across all the plots, the overlap percentage increased markedly from 15.1 ​% in the original images to 64.3 ​% in the corrected images. These results highlight the effectiveness of the CoF-CC method in improving the color accuracy of UAV imagery.

### 3.4 Rice field color quantification and rice maturity prediction

**Fig. 7:**
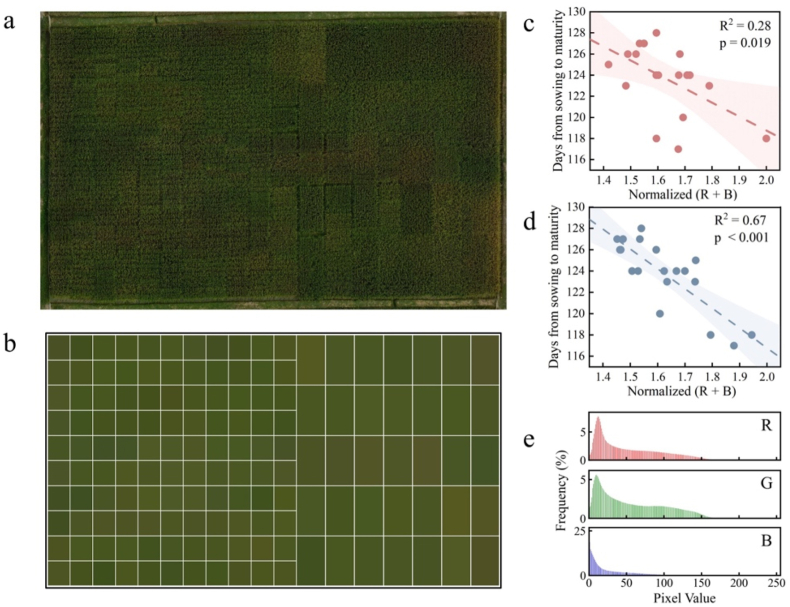
(a) Color-corrected orthomosaic of the experimental field. (b) Visualization of the average canopy color extracted from each rice plot. (c, d) Normalized (R ​+ ​B) values plotted against maturity days for original and color-corrected images, respectively. (e) RGB histograms for a representative color-corrected plot.

Following the generation of the color-corrected field orthomosaic, quantitative color values were extracted and summarized for each individual rice plot (Fig. 7a–b). The CoF-CC method effectively removes background noise caused by varying imaging sensors and lighting conditions, enabling clear differentiation of rice canopy color among breeding materials, which directly reflects their genetic variation across growth stages. Further attempts to determine the correlation between plot canopy color and rice maturity date revealed no significant relationship when the original image canopy color was used, as shown in Fig. 7c (R^2^ ​= ​0.28; p ​= ​0.019). However, the canopy colors derived using the CoF-CC method exhibited a significant linear correlation with the rice maturity date (R^2^ ​= ​0.67, p ​< ​0.001) (Fig. 7d). The markedly enhanced correlation between color and rice maturity dates underscores the importance of standardized data in agricultural remote sensing monitoring.

## 4 Discussion

### 4.1 Addressing illumination variations in open-field RGB remote sensing

**Fig. 8:**
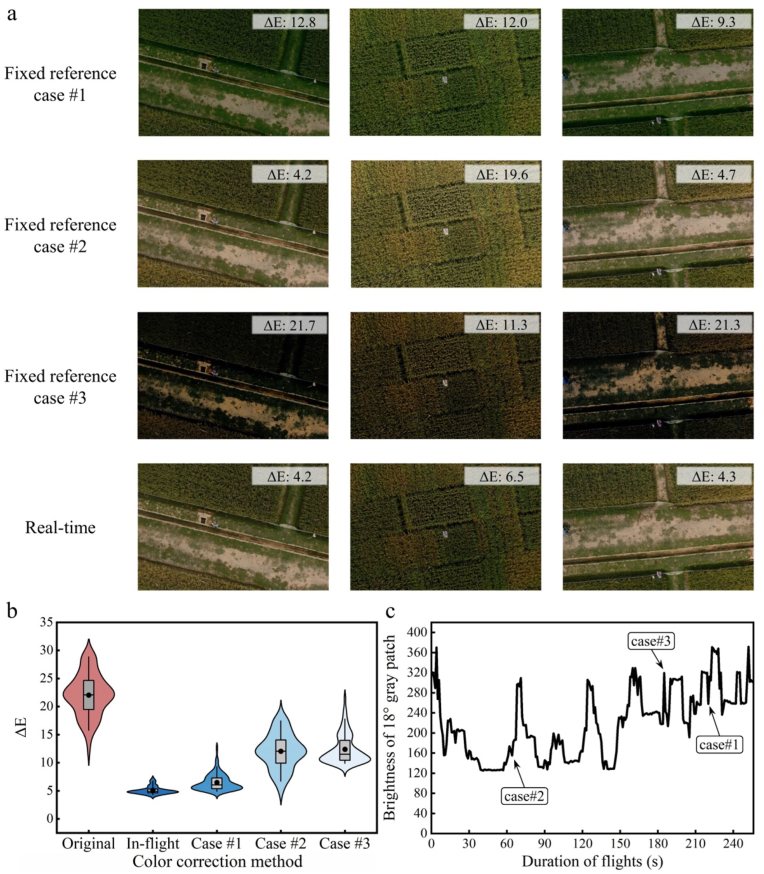
(a) Examples of color-corrected images generated using the proposed in-flight method compared with three randomly selected images from the flight mission as fixed references. The ΔE values indicate the average ΔE of all ColorChecker patches in each image. (b) Comparison of the color correction performance between the proposed in-flight method and the three selected fixed reference images. The distributions show the ΔE values of ColorChecker patches across all the images captured during the flight. (c) Variations in illumination intensity, represented by the brightness values of the 18° gray patch across all original images captured during the flight. Annotations indicate the time points at which the selected fixed reference images were acquired.

The primary advantage of the CoF-CC method lies in its ability to ensure that both the ColorChecker and the crop canopy are captured under identical lighting conditions during UAV flights, effectively mitigating noise caused by dynamic lighting variations. This approach provides improved correction accuracy and robustness compared with the conventional method of using a single, fixed-position ColorChecker for correction. In a previous study, Tocci et al. [17] conducted 30 random field orthomosaic reconstructions across six regions over three days, revealing significant errors in color measurements caused by illumination variations. While implementing color correction using the conventional method of imaging a single, fixed-position ColorChecker during the flight partially reduced these errors, it failed to effectively address within-flight illumination fluctuations. The performance of the conventional technique was also simulated in our study by performing uniform color correction using single ColorCheckers as fixed references. Repeated simulations conducted individually on three ColorCheckers randomly selected along the flight mission exhibited varied correction performances. Although each reduced the color error in the original images to different degrees, all the methods were outperformed by the proposed in-flight method (Fig. 8a–b). The relatively lower color error observed in fixed reference case #1 than in cases #2 and #3 could be attributed to the fact that its ColorChecker ΔE value was the closest to the overall average calculated across all the images, whereas the other two exhibited significant deviations; this is also reflected in the corresponding illumination strength at the respective acquisition times under substantial variations in lighting conditions (Fig. 8c). When comparing fixed reference case #1 with the in-flight correction method, although the color difference of ΔE was higher by only 1.41, greater variation was observed, indicating poorer correction consistency. Additionally, this optimal scenario with a single ColorChecker exists only in theoretical simulations, as lighting conditions are extremely unpredictable under dynamic environments, making it almost impossible to acquire reference images at the ideal time.

In some other reports, efforts were focused on improving color consistency among images captured within the same flight. Xia et al. [20] proposed a color consistency correction method using a parameterized spline curve to dynamically address color discrepancies in image mosaicking. Huang et al. [19] introduced an automatic semantic correspondence search to identify reference regions and establish color mapping, effectively transferring color characteristics from the reference to the target image and mitigating color discrepancies caused by lighting or sensor variability. While the improvements in color consistency achieved in these reports were valuable in applications such as underwater archaeology, cultural heritage preservation, and outdoor architectural reconstruction [37,38], the accuracy of color measurements cannot be guaranteed; thus, these methods cannot be adopted for applications such as plant color quantification. Additionally, Abdalla et al. [18] trained an mCNN model using ColorChecker-calibrated reference images to learn residuals between raw and corrected images, ensuring intra-flight color consistency and improving reference color accuracy. However, relying on preselected reference images limits the ability of this method to fully address dynamic lighting variations.

To summarize, existing methods can reduce discrepancies in image color but fail to achieve precise correction for datasets derived from long-duration flights, whereas the proposed in-flight method effectively addresses environmental lighting variations. Notably, the necessity of in-flight color correction decreases under stable lighting conditions, such as on clear, sunny days. However, such weather conditions can be rare and unpredictable and vary across different regions. The ability of the proposed in-flight correction method to adapt to various weather conditions is valuable because it significantly enhances data fidelity and mission efficiency in UAV-based RGB remote sensing and promotes the standardization of plant color quantification in diverse field applications, such as field monitoring and crop breeding.

### 4.2 Investigations on the CCM method

During UAV imaging, variations in lighting conditions often result in differences in image quality. To address this, cameras automatically adjust exposure parameters based on global metering. However, as global metering accounts for the exposure of all objects within the frame, it cannot prioritize or optimize exposure specifically for ColorChecker, potentially causing overexposure of highly saturated color patches in ColorChecker. Consequently, a varying number of color patches were removed from our analysis. In this regard, Sunoj et al. [29] evaluated color correction performance using the CCM method by progressively incorporating 1 to 24 color patches from ColorChecker, and their findings indicated that correction accuracy stabilized when a minimum of nine color patches was utilized. Similar conclusions can be drawn from our experiments. As illustrated in Fig. S4, increasing the number of color patches used for correction gradually improved the ΔE values. However, the improvement margin decreases when more than 12 patches are utilized, with the difference in ΔE compared with using all 24 patches remaining within 10 ​%. These findings indicate that a few overexposed patches can be tolerated without significantly compromising color correction accuracy. Furthermore, since crop reflectance is generally lower than that of highly saturated ColorChecker patches, future efforts could explore the customization of color charts by reducing the saturation of those patches to minimize the risk of overexposure.

Both linear CCM methods and nonlinear approaches can map ColorChecker values to a standard color space. Nonlinear methods, such as the approach used by Han et al. [39], are better suited for capturing the nonlinear relationships between different color channels, making them particularly effective for multispectral cameras with broader spectral ranges, such as RGBN cameras. In this study, we also tested several of these algorithms and reported that they achieved better correction performance for the 24 ColorChecker patches (Fig. S5). However, noticeable color distortions were observed in the rest of the image; this is because the color distortions in RGB channels are caused primarily by linear errors introduced by the device and lighting conditions. When more complex nonlinear methods are used, overfitting may occur, resulting in poor correction performance for colors outside the ColorChecker patches.

### 4.3 Practical value of accurate color measurements in agriculture

Crop color is a vital, noninvasive indicator of plant health, growth stages, and overall performance, as it reflects physiological changes, nutrient levels, and biotic or abiotic stresses. Dynamic color assessment in the field enables breeders and farmers to make timely decisions on agronomic inputs, such as fertilization, irrigation, and pest management, while also guiding optimal harvest timing to maximize yield, quality, and market value. For instance, Jia et al. [40] utilized a digital camera to record green light reflectance from a winter wheat canopy to assess nitrogen content. Similarly, Tao et al. [41] developed a smartphone application based on a customized color chart to detect the color levels of rice leaves and infer their nitrogen content. Furthermore, as fruit color is a critical indicator of ripeness and quality grading, Motonaga et al. [42] designed a standardized fruit color chart and an image analysis system based on color scales to classify fruits from the immature to mature stages. In such applications, field-based *in situ* measurements are preferred because they minimize the errors associated with color changes that may occur when plant tissues, such as leaves or other organs, are removed for offsite analysis. Additionally, a common aspect among these studies is their reliance on either reflectance from active light sources or side-by-side reference color charts to obtain quantitative color values. While achievable on the ground, these conditions are challenging in UAV remote sensing.

The CoF-CC method, which uses dual-UAV cooperative flight, effectively addresses this challenge and represents a significant advancement in facilitating high-throughput color measurements by UAVs while ensuring the preservation of color accuracy. Notably, a threshold ΔE value of 4.4 was proposed by Uroz et al. [43] as the limit of systematic color differences perceivable by the human eye in the printing industry, serving as a reference for acceptable color error to be targeted. In our results, the ΔE value for ColorChecker was 5.1, while that for the leaves was 5.0. These findings demonstrate that the color accuracy achieved with our method is comparable to that of human vision and is adequate to meet the quantification requirements of most color measurement applications.

Accurate color measurement is essential in various agricultural applications, not only as a critical target in breeding but also as a valuable indicator of crop physiological status and growth conditions. In our prediction of rice maturity dates, the selection of normalized (R ​+ ​B) as the primary color indicator was based on the strong absorption of red (R) and blue (B) light by chlorophyll [[44], [45], [46]]. In plots with high chlorophyll content, which is typically associated with later-maturing rice, the absorption of the R and B bands is stronger, resulting in lower reflected values and consequently lower normalized (R ​+ ​B) values. Conversely, earlier-maturing plots, with lower chlorophyll content at the time of imaging, exhibit less absorption and higher reflection in the R and B bands, leading to higher normalized (R ​+ ​B) values. The lack of correlation observed when colors from the original images were used suggests that the true differences in plot canopy colors were obscured by dynamic lighting conditions. In contrast, these differences became apparent after color correction was applied, demonstrating the significant potential of our method for precise and reliable monitoring of crop physiology in both production and breeding programs.

### 4.4 Limitations and future work

Although the CoF-CC method has proven effective for image color correction, it still faces challenges in efficiently segmenting target boundaries (e.g., leaves, flowers, or fruits) within the images. Accurate color measurements for specific targets require separation from the background, a process that can be both time-consuming and labor-intensive. In this study, manual labeling was used to extract leaf areas, which was not only tedious and subjective but also prone to inconsistencies. To address this limitation, integrating computer vision techniques for automatic target identification could be a valuable addition to the workflow. Successful applications in this domain include panicle detection in rice, bud detection in tea, and weed detection in soybeans [[47], [48], [49]]. By combining automatic target identification with color measurement, a fully automated pipeline for plant target color analysis can be developed, paving the way for more efficient and scalable phenotyping practices.

Another issue caused by the complicated structure of the plant canopy is light occlusions, resulting in a substantial degree of illumination variation at a relatively fine scale at the plant or organ level. When light occlusion is high, which usually occurs between tall plant bodies, it can be difficult to distinguish plants from soil. Even if the plant structure can be recognized, its color is usually strongly biased because of insufficient illumination. In this study, canopy color estimation was performed by averaging all pixels within the plot area. However, the overall mean may be influenced by the darker pixels from the lower canopy layers, potentially introducing bias. Future research is needed to address this issue and refine strategies for summarizing canopy color from images of complex crop fields. Leveraging artificial intelligence or foundation models could automate the identification of high-quality leaf imaging regions while excluding areas affected by angles, shadows, or specular reflection [50]; this ensures that the selected leaf pixels more accurately represent true color characteristics, enhancing the robustness and precision of color correction in agricultural applications.

In addition, the reflective properties of leaves differ significantly from those of the ColorChecker surface, which is a flat, smooth standard designed to approximate an ideal Lambertian plane and exhibits consistent reflectance under varying illumination angles [51]. During dual-UAV collaborative flights, ColorChecker remains horizontal, avoiding angular variations. In contrast, crop organs such as rice leaves, whose waxy surfaces, veins, and varying orientations, exhibit complex bidirectional reflectance distribution function properties [52], including specular and anisotropic reflection, as well as shadowing effects; this is evidenced by the evaluation of the RGB channel histograms summarized for the crop canopy, which revealed a wide distribution of color values, with no distinct peaks serving as clear color indicators (Fig. 7e). Consequently, future challenges in color measurement not only include achieving precise color correction but also developing methodologies for accurately extracting color information from crop canopies. Future research may benefit from integrating leaf-specific optical properties with advanced remote sensing [53]. By incorporating anisotropic reflective behavior, multiangle, multispectral, or multitemporal data can be used to construct detailed leaf optical models, capturing BRDF and structural features such as angular distribution and wax layer reflectance for improved correction models [54].

In open-field RGB remote sensing, significant noise arises from the complex interactions between sensor characteristics, imaging parameters, hardware platforms, and the diverse sampling conditions encountered across experiments conducted by various research teams [11]. Existing standardization and calibration approaches have been reported to address similar challenges, such as those in thermal and spectral image data [55,56]. Similarly, the CoF-CC method proposed in this study aims to enhance the consistency and accuracy of RGB data by leveraging cooperative UAV flights. Continued advancements in this field are essential for further improving data sharing and reproducibility and fostering progress in agricultural research and decision-making.

## 5 Conclusions

This study demonstrated the strong performance of the CCM method across six different cameras, with a 66.1 ​% reduction in the average color error (ΔE) from 12.4 to 4.2 and a 70.2 ​% reduction in the intracluster distance from 13.2 to 3.9. Building on this, a dual-UAV in-flight color correction method was developed to address the challenges associated with achieving precise and consistent crop color measurements under dynamic field lighting conditions. The CoF-CC method reduced color errors by an average of 73.6 ​% for color patches and 72.7 ​% for rice leaf samples; comparisons with existing methods further highlight its superior ability to handle environmental illumination variations, ensuring reliable and robust color measurements for high-throughput UAV-based crop phenotyping. Furthermore, the application of this method in rice breeding plots revealed significant correlations between corrected plot colors and maturity dates, underscoring its utility in field-based phenotypic analyses. Overall, the proposed method provides a reliable and scalable solution for accurate color measurement in precision agriculture, enhancing data fidelity and supporting efficient breeding and agricultural decision-making under diverse environmental conditions.

## Author contributions

X.L. and J.X. contributed to the experimental design, model construction, model validation, and manuscript writing. J.Y. provided the experimental materials and facilities. J.Z. edited the manuscript. H.C. supervised the experiments at all stages and revised the manuscript. All the authors read and approved the final manuscript.

## Funding

This work was funded by the 10.13039/100019767International S&T Cooperation Program of China (2024YFE0115000), the National Key R & D Program of China (2021YFD2000104), the 10.13039/501100001809National Natural Science Foundation of China (32371985), and the Fundamental Research Funds for Central Universities (226-2022-00217).

## Data availability

The UAV image datasets used in this study are openly available in the GitHub repository at https://github.com/GaryLXQ/RiceUAVImageData.

## Declaration of competing interest

The authors declare that they have no known competing financial interests or personal relationships that could have appeared to influence the work reported in this paper.
