# Supplementary material for: Precise Image Color Correction Based on Dual Unmanned Aerial Vehicle Cooperative Flight
Source: Plant Phenomics. 2025 Sep 5;7(3):100101. doi: 10.1016/j.plaphe.2025.100101 (PMC12710031; doi:10.1016/j.plaphe.2025.100101)
Supplement: Multimedia component 1 [file mmc1.docx]

**Supplementary materials**

**
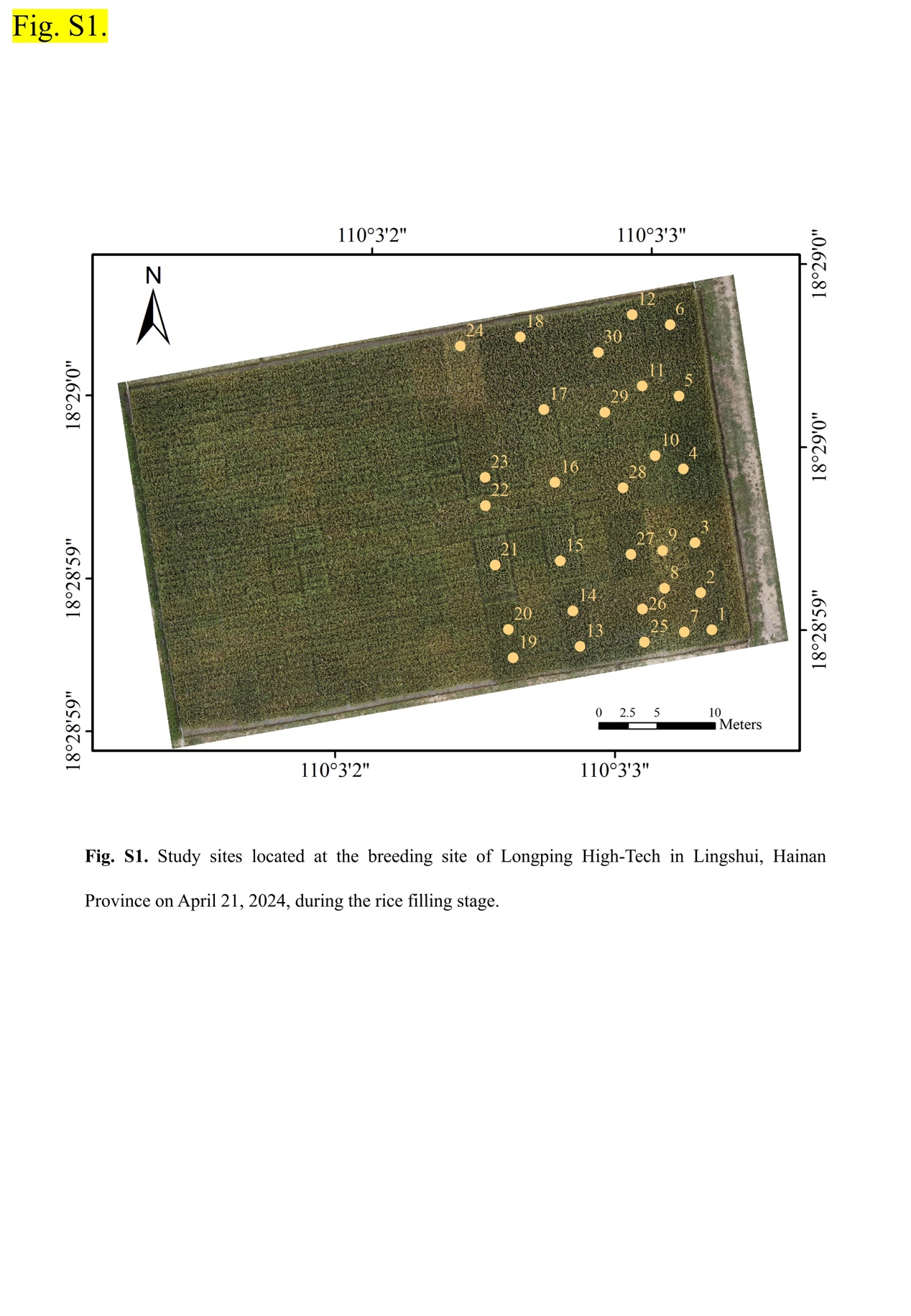
**

Fig. S1. Study sites were located at the breeding site of Longping High-Tech in Lingshui, Hainan Province, on April 21, 2024, during the rice filling stage. Yellow points indicate the precise RTK positions of the leaf samples.


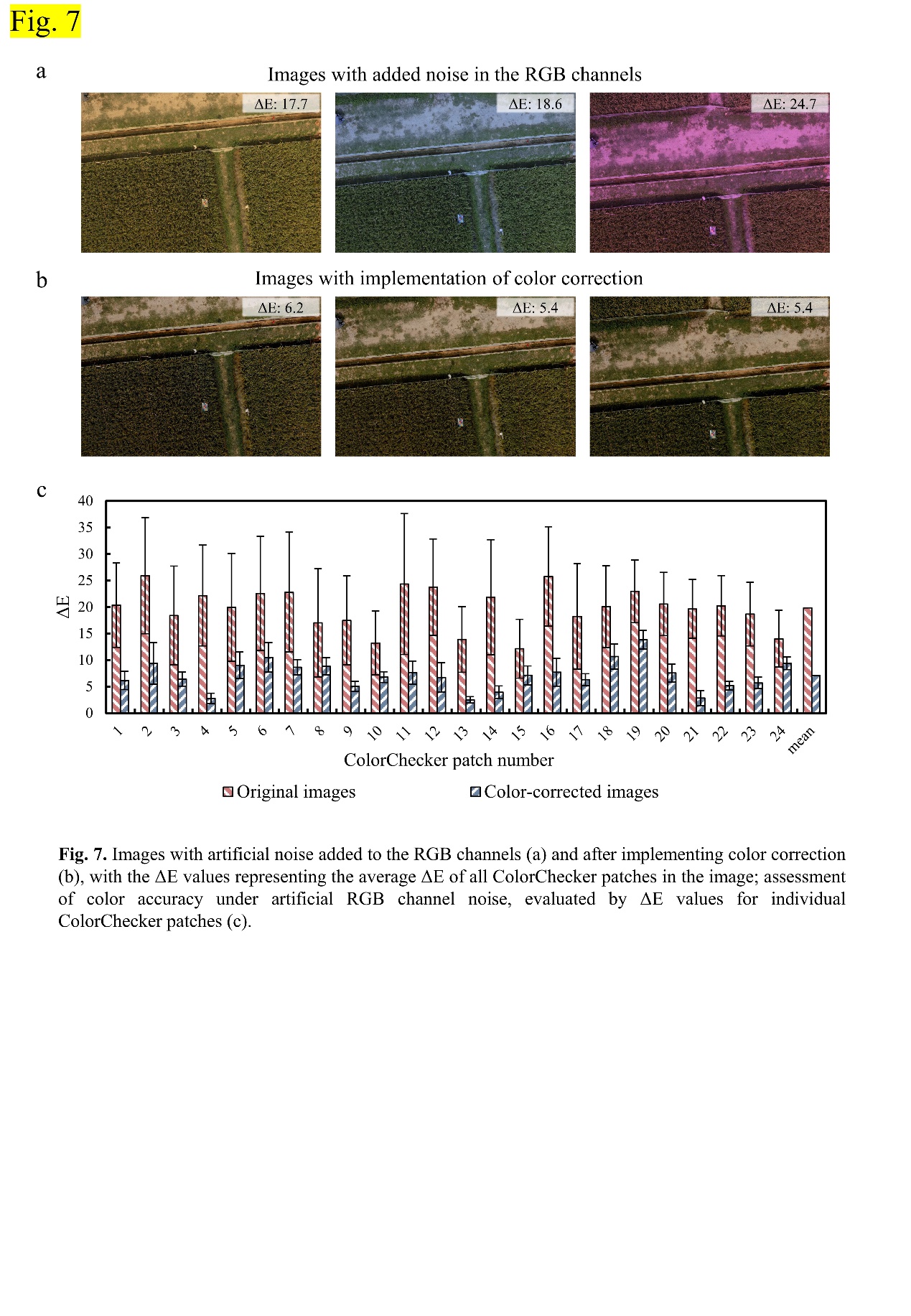


Fig. S2. (a) Images with added noise in the RGB channels (b) and after implementing color correction, with the ΔE values representing the average ΔE of all ColorChecker patches in the image. (c) Assessment of color correction accuracy in images with added noise, evaluated by ΔE values for individual ColorChecker patches.

**
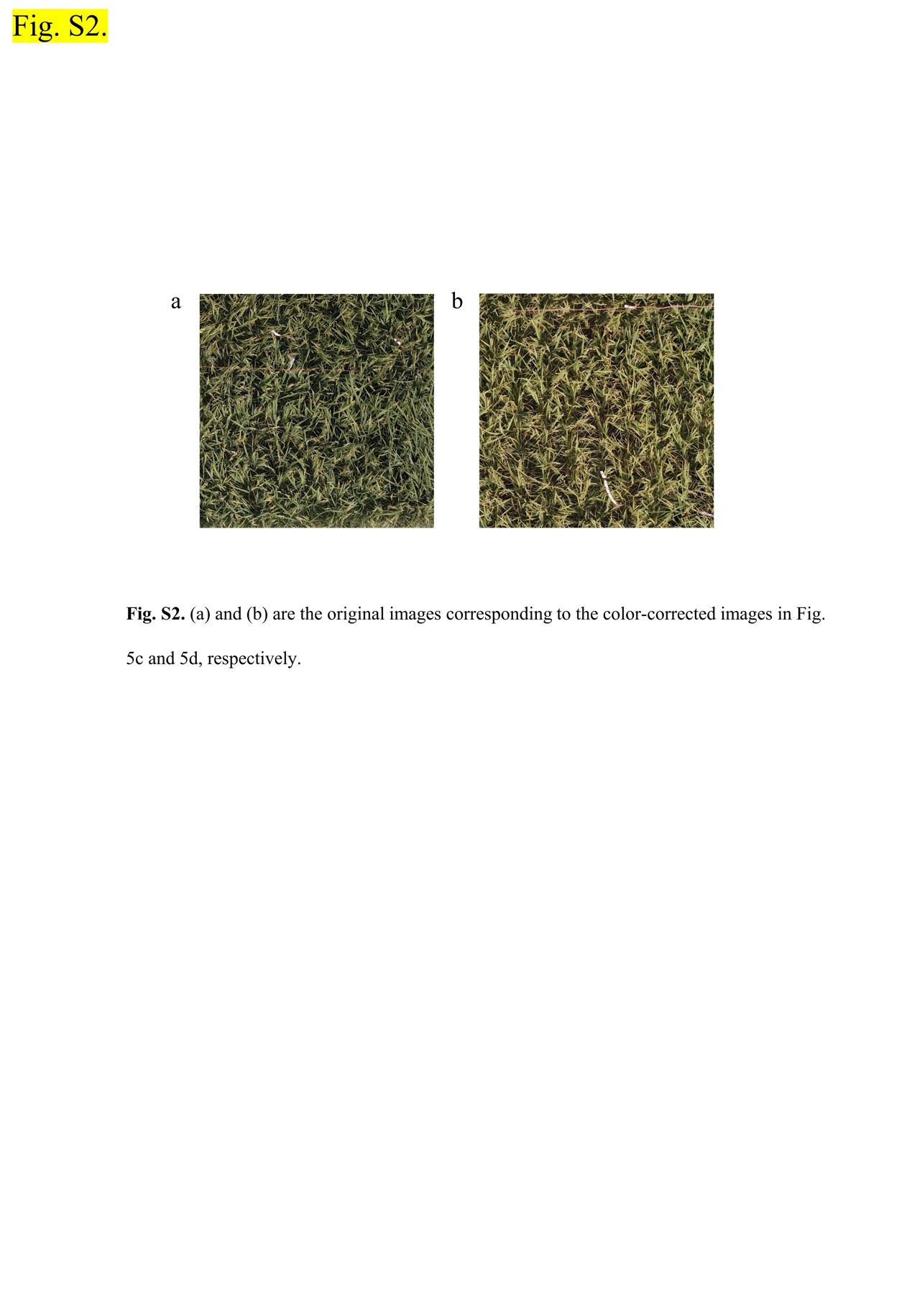
**

Fig. S3. (a) Original image corresponding to the color-corrected image in Fig. 5c. (b) Original image corresponding to the color-corrected image in Fig. 5d.


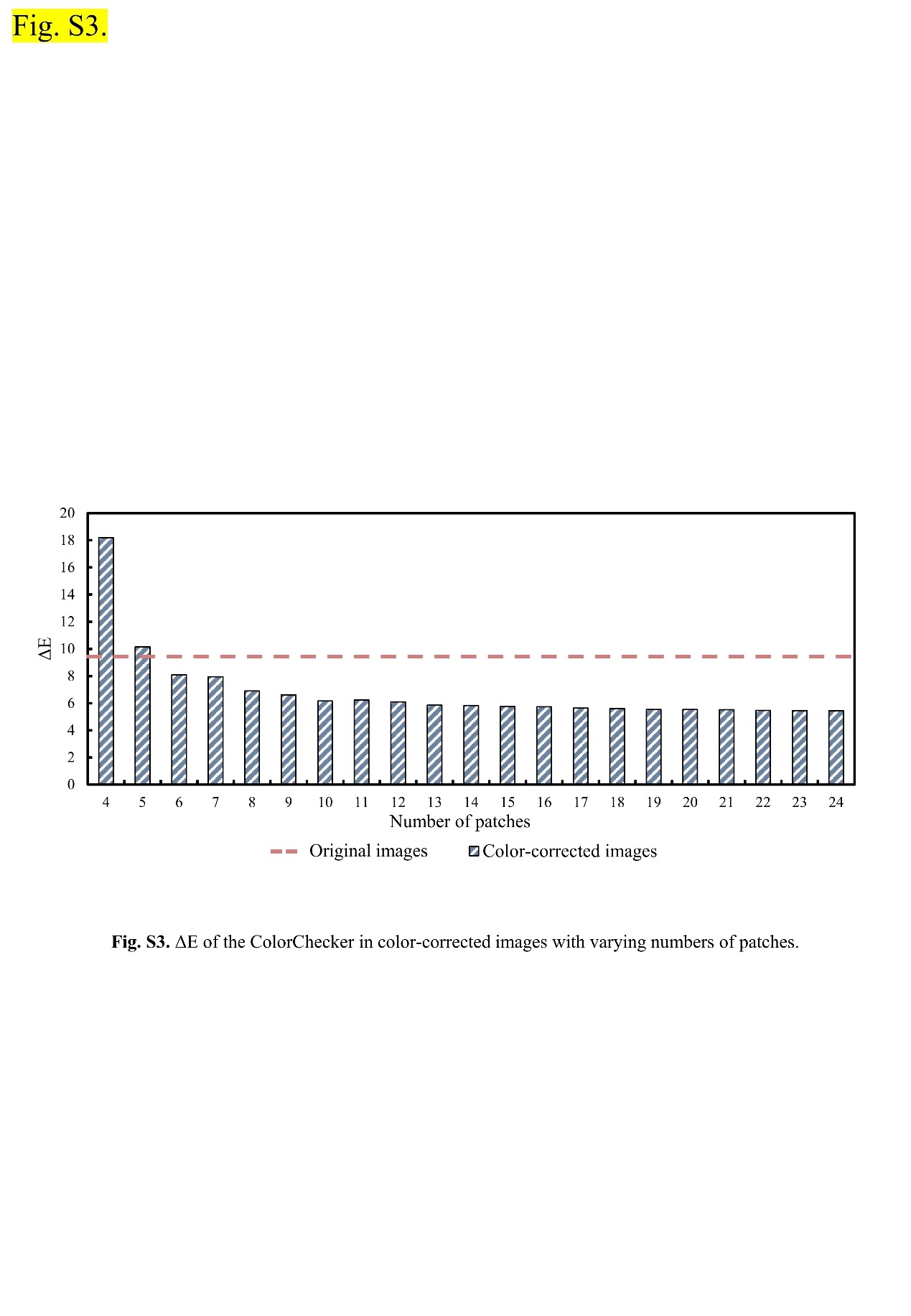


Fig. S4. ΔE of ColorChecker in color-corrected images with varying numbers of patches.


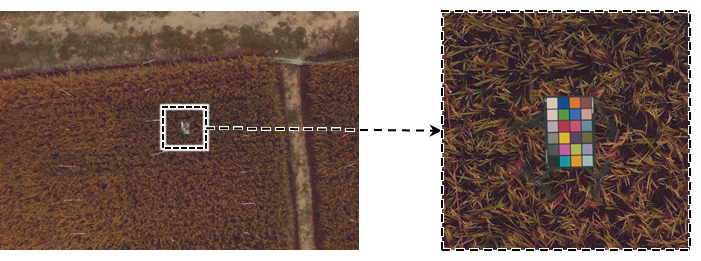


Fig. S5. Nonlinear correction using polynomial transformation

Table S1. Average RGB values of the 24 ColorChecker patches captured at different heights

| Height (m) | Average R | Average G | Average B |
| --- | --- | --- | --- |
| 6 | 224.08 | 225.91 | 220.50 |
| 7 | 223.83 | 225.45 | 218.60 |
| 8 | 223.67 | 225.33 | 219.67 |
| 9 | 217.08 | 219.50 | 216.08 |
| 10 | 223.50 | 225.25 | 219.38 |
